# Poison of Snakes in Australia

**Published:** 1845-06

**Authors:** 


*Poison of Snakes in Australia.*—"The poison of the generality of Australian snakes appears to act differertly from that of the rattlesnake of America, or the viper of England; for whereas the poison of the latter species creates immediately a marked effect on the punctured wound, causing violent swelling, intense pain, and a yellow or livid hue over the surface, the bite of Australian snakes does not cause much pain or inflammation in the wound itself, but seems principally to affect the whole nervous system, rapidly causing the patient to fall into a comatose state. In this respect, the poison resembles that of the asp of Egypt.—Hodskinson's *Australia.* T. R. B. *in the Am. Jour Med.*

